# Effects of Vitamin D-Deficient Diet on Intestinal Epithelial Integrity and Zonulin Expression in a C57BL/6 Mouse Model

**DOI:** 10.3389/fmed.2021.649818

**Published:** 2021-08-03

**Authors:** Chun-Yan Yeung, Jen-Shiu Chiang Chiau, Mei-Lein Cheng, Wai-Tao Chan, Chuen-Bin Jiang, Szu-Wen Chang, Chia-Yuan Liu, Ching-Wei Chang, Hung-Chang Lee

**Affiliations:** ^1^Department of Pediatric Gastroenterology, Hepatology and Nutrition, MacKay Children's Hospital, Taipei, Taiwan; ^2^Department of Medical Research, MacKay Memorial Hospital, Taipei, Taiwan; ^3^Department of Medicine, MacKay Medical College, New Taipei City, Taiwan; ^4^Department of Hepatology and Gastroenterology, MacKay Memorial Hospital, Taipei, Taiwan

**Keywords:** vitamin D, gut integrity, tight junction, zonulin, leaky gut

## Abstract

**Background and Aims:** Vitamin D (VD) plays an important role not only in mineral balance and skeletal maintenance but also in immune modulation. VD status was found correlated with the pathophysiology and severity of inflammatory bowel diseases and other autoimmune disorders. Epithelial barrier function is primarily regulated by the tight-junction (TJ) proteins. In this study, we try to establish an animal model by raising mice fed VD-deficient diet and to investigate the effects of VD-deficient diet on gut integrity and zonulin expression.

**Methods:** Male C57BL/6 mice were administered either VD-deficient [VDD group, 25(OH)_2_D_3_ 0 IU/per mouse] or VD-sufficient [VDS group, 25(OH)_2_D_3_ 37.8 IU/per mouse] special diets for 7 weeks. Body weight and diet intake were recorded weekly. Serum VD levels were detected. After sacrifice, jejunum and colon specimens were collected. The villus length and crypt depth of the jejunum as well as mucosa thickness of the colon were measured. Various serum pro-inflammatory cytokines and intestinal TJ proteins were assessed. The serum level of zonulin and the mRNA expression of jejunum zonulin were also investigated.

**Results:** We found that mice fed a VDD diet had a lower serum level of VD after 7 weeks (*p* < 0.001). VDD mice gained significant less weight (*p* = 0.022) and took a similar amount of diet (*p* = 0.398) when compared to mice raised on a VDS diet. Significantly decreased colon mucosa thickness was found in VDD mice compared with the VDS group (*p* = 0.022). A marked increase in serum pro-inflammatory cytokine levels was demonstrated in VDD mice. All relative levels of claudin (CLD)-1 (*p* = 0.007), CLD-3 (*p* < 0.001), CLD-7 (*p* < 0.001), and zonulin-1 (ZO-1, *p* = 0.038) protein expressions were significantly decreased in the VDD group when compared to the VDS group. A significant upregulation of mRNA expression of jejunum zonulin (*p* = 0.043) and elevated serum zonulin (*p* = 0.001) were found in the VDD group.

**Conclusions:** We successfully demonstrated that VDD could lead to impaired barrier properties. We assume that sufficient VD could maintain intestinal epithelial integrity and prevent mucosal barrier dysfunction. VD supplementation may serve as part of a therapeutic strategy for human autoimmune and infectious diseases with intestinal barrier dysfunction (leaky gut) in the future. To our knowledge, this is the first study to demonstrate that VDD could lead to a significant upregulation in mRNA expression of the jejunum zonulin level and also a marked elevation of serum zonulin in a mouse model.

## Introduction

In addition to its principal function as a calcium regulator in facilitating the absorption and metabolism of calcium and bone health, vitamin D (VD) can affect cell and tissue morphology. VD also has immune regulatory functions and contributes to the homeostasis in the body (1). VD protects the gut barrier by regulating tight-junction (TJ) proteins and inhibiting intestinal apoptosis. VD deficiency (VDD) is associated with a number of diseases, such as allergic diseases, inflammatory bowel disease (IBD), and autoimmune disorders (2–4). In addition, serum VD levels are inversely correlated with the degree of non-alcoholic steatohepatitis and fibrosis in children with non-alcoholic fatty liver disease (5, 6). We have also demonstrated that VD levels are inversely associated with the severity of fibrosis of the native liver in patients after Kasai's portoenterostomy for biliary atresia (7).

The gastrointestinal tract is the largest immunological organ in the body and has a central role in immune homeostasis (8, 9). Epithelial barrier function is primarily regulated by the TJ proteins. VD is involved in the regulation of the epithelial barrier functions (10, 11). VD is an important mediator of intestinal epithelial defenses against infectious agents, and VDD predisposes to severe intestinal injury (12, 13). Mice with simple VDD are susceptible to colitis because of impaired colonic antimicrobial activity and homeostasis of enteric bacteria (14). VDD is related to the high incidence of colorectal cancer, and VD supplementation may inhibit the development of colorectal cancer (15). VD may reverse colorectal cancer through regulating intestinal flora, especially *Akkermansia muciniphila*, and maintaining colon barrier integrity (15).

Mice fed a high-fat and VDD diet have an increased amount of pathogens (*Helicobacter hepaticus*) in the gnawer ileum, but the amount of symbiotic bacteria (*Akkermansia muciniphila*) markedly decreased (16). Epithelial barrier function is primarily regulated by the TJ proteins. We can foresee the translocation of pathogens to intestine once dysfunction of TJ occurs. These functions require a highly organized TJ morphology which may be modified by VD supplementation. Besides, zonulin is the eukaryotic counterpart of the *Vibrio cholerae* zonula occludens toxin (17). Human zonulin is identical to prehaptoglobin-2 and binds to the epidermal growth factor receptor and protease-activated receptor 2 in the intestinal epithelium. This complex initiates the phosphorylation of zonula occludens proteins and leads to the small intestine's TJ disassembling (18). According to previous research studies, zonulin is the only measurable blood protein that reflects the intestinal permeability, and increased zonulin level is considered to be a marker of impaired intestinal barrier (19, 20).

Investigators in most studies look at the effect of VD on the recovery of intestinal injury in infected mouse models (21–23). They seldom investigate the roles of VD on the integrity of gut morphology and function of TJ proteins. We hypothesized that VD was involved in the regulation of the epithelial barrier functions and VDD might predispose mice to intestinal injury, since zonulin is the only measurable blood protein that reflects the intestinal permeability and increased zonulin level should be observed in mice fed with a VDD diet. In this study, we try to establish an animal model by raising mice fed a VDD diet in order to elucidate the roles of VD in gut morphology and barrier functions. We also investigate the enterocyte microstructures, inflammatory cytokines, and TJ protein expressions in this mouse model. The serum level of zonulin and the mRNA expression of jejunum zonulin were also investigated.

## Materials and Methods

### Animals' Experiment and Ethics Statement

Male C57BL/6 mice (3–4 weeks of age) were used in all experiments and were approved by the Institutional Animal Care and Use Committee (IACUC) of MacKay Memorial Hospital (IACUC number: MMH-A-S-107-026). IACUC has been accredited, approved, and authorized by the government office, Agriculture and Food Agency Council of Agriculture, Executive Yuan, Taiwan. All methods were performed in accordance with the relevant guidelines and regulations in this animal study.

To produce standard VD-3 [25(OH)_2_D_3_] concentrations (1,500 IU/kg diet) in the circulation, mice were fed a ssniff R/M-H diet (E15312-24; VD deficient, normal vitamin D&P, ssniff Spezialdiäten GmbH, Soest, Germany) containing either 37.8 IU supplement/per week based on the consumption of 25.2 g diet/per mouse weekly at the age of 6 weeks (adult mouse) or saline solution by oral gavages once a week (24).

We used cholecalciferol VD-3 [25(OH)_2_D_3_] as the VD diet source in this mouse model study. Body weight and diet intake were recorded weekly. After 7 weeks, the serum 25-hydroxyvitamin D3 [25(OH)_2_D_3_] concentrations were measured in the mice using the ELISA kit. Blood samples were obtained *via* cardiac puncture and were centrifuged to yield serum.

### Cytokine Analysis

The serum (50 μl) was analyzed by the Bio-Plex Pro^™^ Mouse Cytokine Multiplex Panel kit (Bio-Rad Laboratories Inc., Hercules, CA, USA). Targets of cytokines included IL-1β, IL-6, IL-10, IL-12, IFN-γ, MCP-1, and TNF-α. Extracted serum utilized the Bio-Plex 200 system (Luminex Co., Austin, TX, USA). The tissues lysates were extracted from the jejunum.

### Immunofluorescent Localization of TJ Proteins

The location of TJ proteins was studied using immunohistochemistry. Samples of jejunum were embedded in paraffin, cut into 3-μm sections, mounted on slides glasses, and deparaffinized by standard protocols. For antigen retrieval, the tissues were treated with Tris/EDTA solution buffer (10 mM Tris, pH 9; 1 mM EDTA). Incubation with primary rabbit anti-ZO-1 antibody, anti-claudin (CLD)-1, anti-CLD-3 (Sigma, Merck, Germany), anti-CLD-7 (Abcam, Cambridge, UK), and mouse anti-occludin (OCDN) (Life Technologies, Carlsbad, CA, USA) was conducted at 4°C overnight followed by PBS washing. DyLight 488-conjugated anti-rabbit antibody and DyLight 549-conjugated anti-mouse antibody (Jackson, Bar Harbor, ME, USA) were used as secondary antibodies. Then, three more washes were performed. The fluorescence of the TJ proteins (ZO-1 and CLD-1) was examined using a confocal microscope (MRC 600; Olympus, Tokyo, Japan) with a krypton argon laser. The fluorescence of the TJ proteins (CLD-3, CLD-7, and OCDN) was examined using a fluorescence microscope (AX10, Zeiss, Jena, Germany). The images collected had an optical thickness of 3 microns for the jejunum. The images shown represented a projection of the sections made for each villus.

### Histological Analysis

Histological analysis was performed according to the standard protocol that was published in the literatures (25, 26). Briefly, the tissues of jejunum and colon were processed and fixed in 10% buffered neutral formalin. The tissues of jejunum and colon were processed and fixed in 10% buffered neutral formalin. Then, the tissues were further processed and embedded into paraffin. The samples were cut into 3-mm-thick sections. All sections were deparaffinized and stained with hematoxylin and eosin (H&E) according to standard procedures. The sections were photographed by using a TissueFAXS automatic scanning system, captured by a digital camera, and analyzed by HistoQuest software (TissueGnostics, Vienna, Austria). Four mice in each group were sacrificed for parameter determination. Measurements of villus height and crypt depth of the small intestine were determined for whole well-orientated villi and crypts per small intestinal tissue section per mouse, and the values were averaged. We also assessed the colonic specimens for histological changes and mucosa thickness measurements. The villus height, crypt depth, villus height/crypt depth ratio of each jejunal tissue section and the muscular layer and mucosa thickness of each colon tissue were measured to determine if gut integrity was whole and well-oriented.

### Western Blot of TJ Proteins

Jejunum tissues were lysed in an ice-cold lysis buffer including Tris–HCl, NaCl, MgCl_2_, glycerol, NP-40, SDS, aprotinin, leupeptin, PMSF, and pepstatin A and placed on ice for 10 min as described in our previous study (27). The supernatant of lysed samples was collected after centrifugation. The total protein concentration was quantified by BCA protein assay kit (Thermo, USA). The protein was added to an equal volume of 2× Laemmli sample buffer and boiled for 10 min and then run at 8% polyacrylamide gel at 100 V for 1.5 h. The treated protein was transferred to Immunoblot PVDF membranes (Bio-Rad). After overnight blocking (PBS/Tween supplemented with 0.05% non-fat dry milk), blots were incubated with rabbit polyclonal antibodies to CLD-1, CLD-3, CLD-7, OCDN, ZO-1, and GAPDH in 0.05% Tween 20/TBS for 4 h. GAPDH was used as a loading control. The secondary antibodies were horseradish peroxidase conjugated anti-mouse IgG. Immunoreactive bands were visualized by chemiluminescence reagents and exposed to an X-OMAT film. Band densities were determined using XnView software. A ratio of variety TJ proteins to GAPDH in the control band was calculated for each sample.

### Measurement of Serum Zonulin Level and Intestinal Zonulin mRNA Expression

Serum zonulin as a marker of leaky gut was measured by the zonulin competitive ELISA kit (MyBioSource, San Diego, CA, USA). The assay sensitivity was 0.1 μg/ml according to the manufacturer's instructions. Quantitative real time PCR (qRT-PCR) was used to determine the expression of zonulin at the level of mRNA. Pairs of oligonucleotide primers specific to zonulin used in our study included the following (28):

forward primer, 5′-TCATCACGGCGCGCCAGG-3′

reverse primer, 5′-GGAGGTCTAGAATCTGCCCGAT-3′.

As mentioned in our previous study, total RNA was isolated from jejunum specimens using the TRIzol reagent (Invitrogen) and was then used for cDNA synthesis with random hexamers. DNA detection and amplification were also detected by qPCR using an ABI 7500 Fast System v1.4.0 (Applied Biosystems). Gene expression was normalized to the GAPDH expression levels (29).

### Statistical Analysis

The quantitative data were expressed as mean ± standard error (SE) for triplicate measurements. Statistical analyses were performed with an independent *t*-test using SPSS 12.0. Statistical significance was defined as a *p* < 0.05.

## Results

### Effects of VDD on Weight Gain and Diet Intake

**Figure 1:**
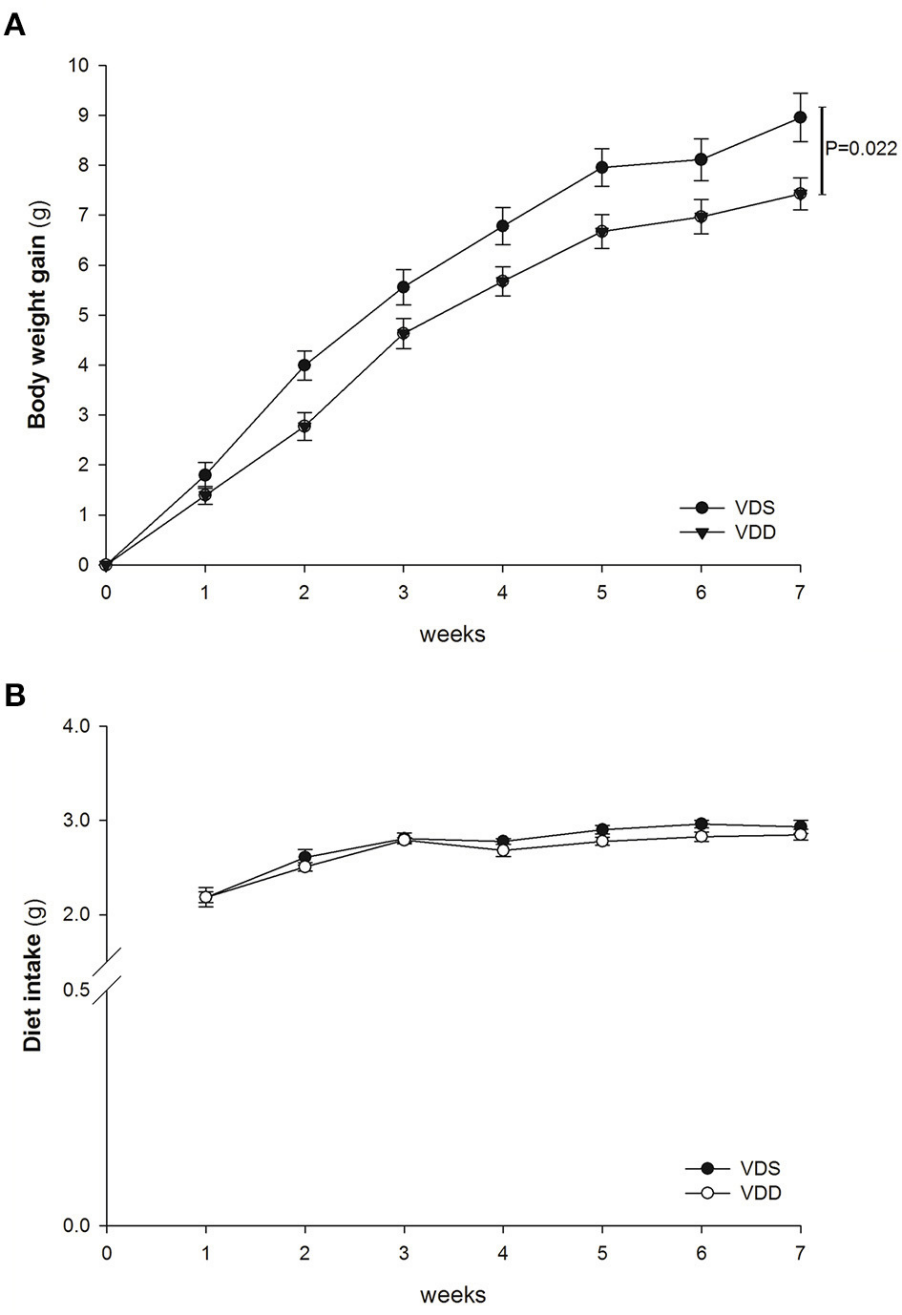
Weight gain and diet intake. The average weight gain and diet intake in both VDD and VDS groups were shown. **(A)** Average body weight gain. VDD mice gained less weight, and a significant difference was found between the two groups (*p* = 0.022 at the 7th week). **(B)** Average amount of diet intake. VDD mice had a similar amount of diet intake compared to the VDS mice during the experiment (*p* = 0.398). The statistical analysis was performed by the independent *t*-test. Five mice in each group for parameter determination.

After completion of the experiment, all mice tolerated well and no animal exhibited signs of marked adverse effects such as bloody stool passage or cachexia. No mortality was noted. The mice were weighed daily, and the results of the two groups were compared. In the beginning of the study, we used male C57BL/6 mice (3–4 weeks of age) in all experiments, so the initial average diet intake amount (2.19 g) was lower than the standard adult level; however, it increased gradually to 2.9 g at the end of the experiment. VDD mice gained significantly less weight (VDD 147.14 ± 2.96% vs. VDS 161.24 ± 4.91%, *p* = 0.022) (Figure 1A) when compared to mice raised on a VDS diet after 7 weeks. The average amounts of diet intake of both groups are shown in Figure 1B. The difference on feed intake did not affect the study result since VDD mice took a similar amount of diet during the experiments when compared to mice raised on VDS diet after 7 weeks.

### VDD Affects the Serum Vitamin D_3_ Level

**Figure 2:**
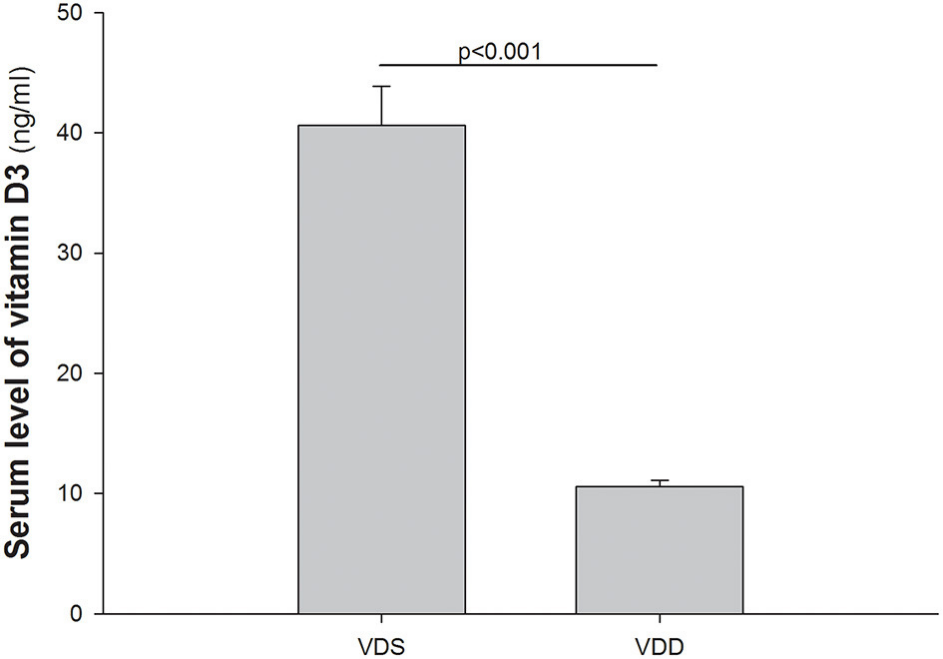
Serum levels of 1,25(OH)_2_D_3_. Serum levels of 25(OH)_2_D_3_ in the VDD and VDS groups are shown. The serum level of 25(OH)_2_D_3_ was 10.45 ± 0.61 ng/ml after 7 weeks in the VDD group while the serum level of 25(OH)_2_D_3_ was 40.62 ± 3.24 ng/ml during the study period in the VDS group (*p* < 0.001). The statistical analysis was performed by the independent *t*-test. Five mice in each group for parameter determination.

The serum levels of VD [25(OH)_2_D_3_] in the VDD and VDS groups are shown in Figure 2. In the VDD group, the serum level of 25(OH)_2_D_3_ was 10.45 ± 0.61 ng/ml after 7 weeks. On the contrary, the serum level of 25(OH)_2_D_3_ in the VDS group was significantly higher (40.62 ± 3.24 ng/ml) than the VDD group during the studied period (*p* < 0.001).

### VDD Causes Intestine Histology and Damage

**Figure 3:**
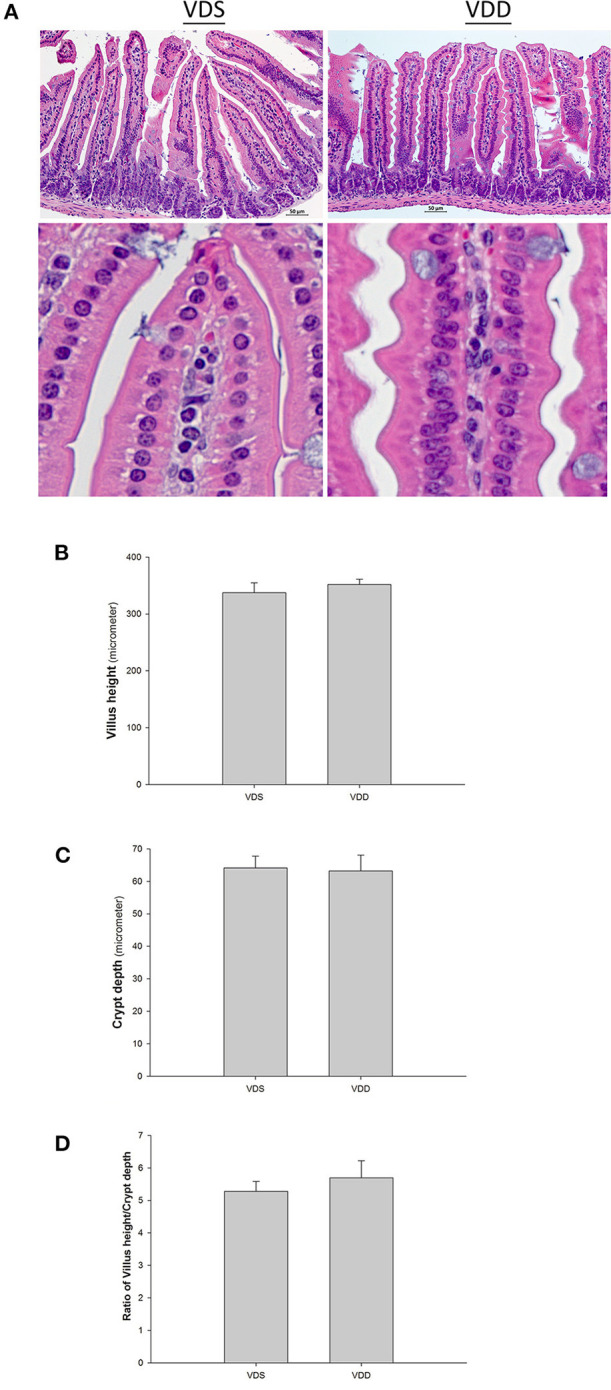
Effect of vitamin D deficiency on the intestinal histology. **(A)** Representative histological images (HE stains) of the jejunum in both groups. VDD mice showed worsened histologic damage with typical irregular edge lining of the jejunal mucosa, distorted enterocytes, and significant inflammatory cell infiltration. Intestinal villi of the mice in the VDS group were more uniform, and inflammatory cell infiltration was absent. Lower photo: magnification 750%. Segments of the jejunum were taken for measurement of the villus height **(B)**, crypt depth **(C)**, and villus height/crypt depth ratio **(D)** per mouse. The levels in villus height, crypt depth, and villus height/crypt depth ratio of the jejunum were compared, and no significant changes were demonstrated between the two groups. Values were represented as mean ± SEM and were analyzed using the independent *t*-test. Four mice in each group sacrificed for parameter determination. More than 15 villi and crypts in each staining of the slice in both groups.

**Figure 4:**
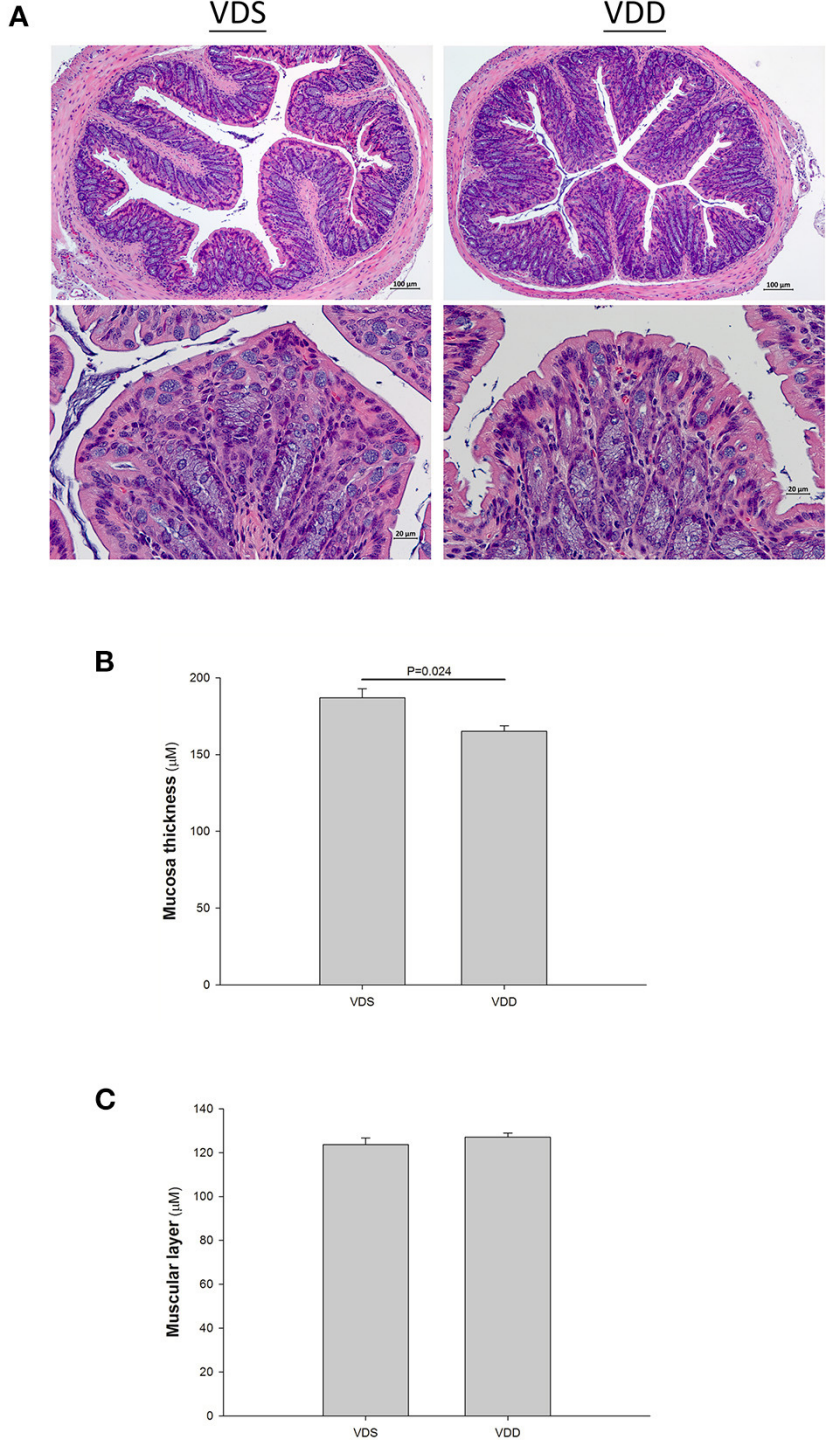
Effect of vitamin D deficiency on the colonic histology. **(A)** Representative histology of colonic specimens with H&E stains in mice. Marked histological damages were found in the colonic specimens in the VDD group. Lower photo: magnification 750%. Segments of colon were taken for measurement of the muscular layer **(B)** and mucosa thickness **(C)**. Both groups had similar thickness of muscular layer (*p* = 0.395). However, VDD mice had significantly decreased colonic mucosa thickness compared to mice raised on VDS diet (*p* = 0.024). Values were represented as mean ± SEM and were analyzed using the independent *t*-test. Four mice in each group sacrificed for parameter determination. More than 15 fields for each staining of the slice in both groups.

The effect of the VDD diet on the intestine histology is shown in Figure 3. The HE stain shows a typical irregular edge and lining of the jejunal mucosa, distorted enterocytes, and significant inflammatory cell infiltration in VDD mouse. However, the intestinal villi of the mouse in the VDS group were more uniform and inflammatory cell infiltration was absent (Figure 3A). We found no significant differences in the villus height level, crypt depth level, and villus height/crypt depth ratio of the jejunum between the two groups (Figure 3B). Besides, we found that the muscular layer thickness of both groups was similar. VDD mice had slightly higher jejunal mucosa thickness compared with VDS mice but did not reach significant difference (data not shown). Similar histological damages were found in the colonic histology in the VDD group, including decreased mucosa thickness, crypt hyperplasia, loss of epithelial integrity, and inflammatory cell infiltration (Figure 4A). Both groups had similar thickness of the muscular layer (Figure 4B). However, VDD mice had significantly decreased colonic mucosa thickness compared with mice raised on VD-sufficient diet (VDD 165.09 ± 3.69μm vs. VDS 187.03 ± 5.80μm, *p* = 0.022) (Figure 4C).

### VDD Affects Intestine TJ Protein Structure

**Figure 5:**
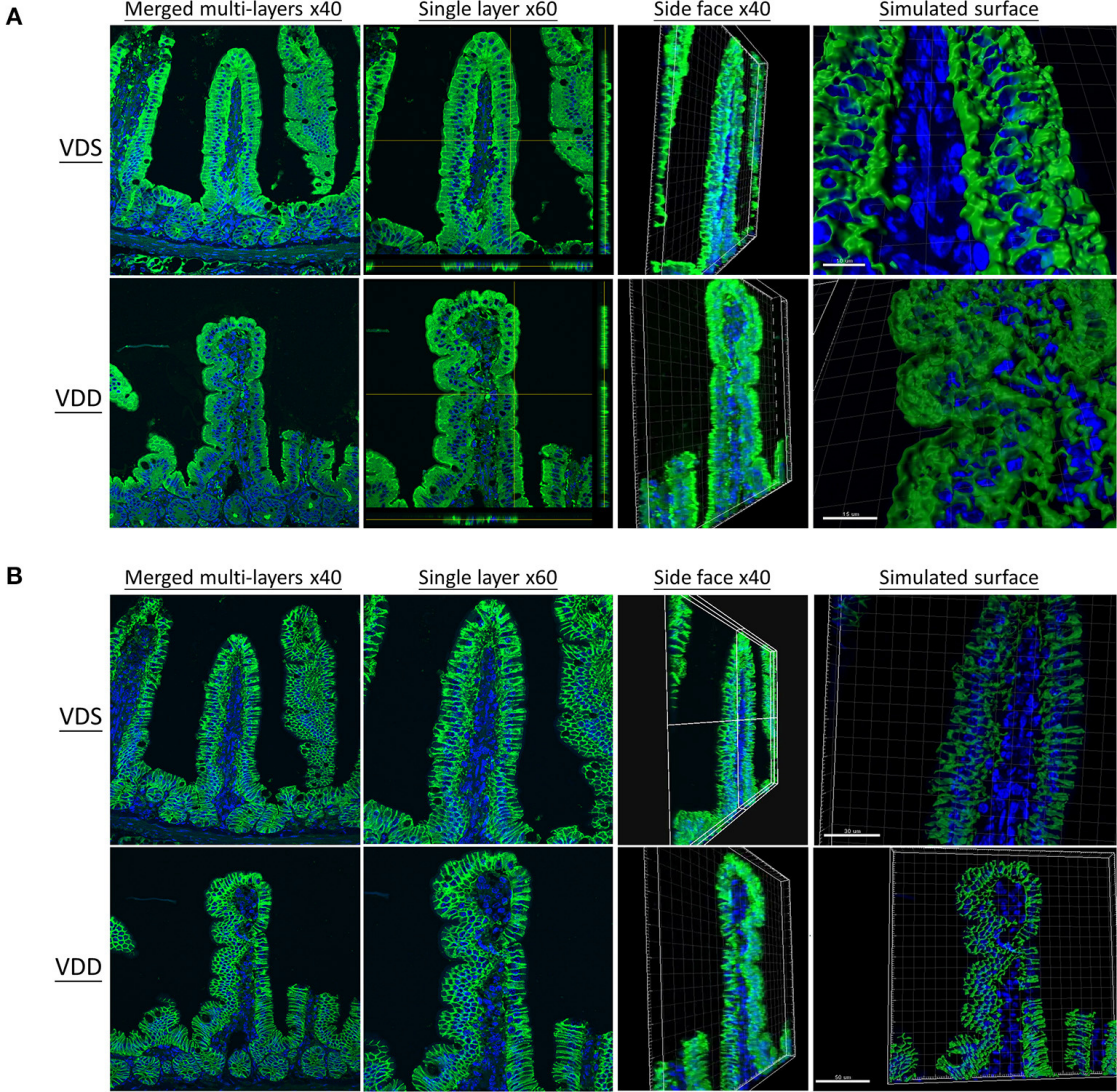
Vitamin D maintains intestinal tight junction expressions in VDD mice by both en face view MPM and reflectance confocal microscopy (RCM) of the jejunum. DAPI was used for nucleus labeling (blue). Front, side face, and simulated surface were displayed. The distribution of tight junctions was visualized by the zonula occludens-1 (ZO-1) fusion protein (DyLight 488, green) **(A)** and claudin-1 (CLD-1) (DyLight 488, green) **(B)**. The fluorescence line was clear and sharp in the VDS group but blurry, cloudy, and irregular in the VDD group at ZO-1 and CLD-1 distributions. Cell nuclei (blue dot) were found located at the baseline of enterocytes in the VDS group but irregular arrangement in the VDD group.

**Figure 6:**
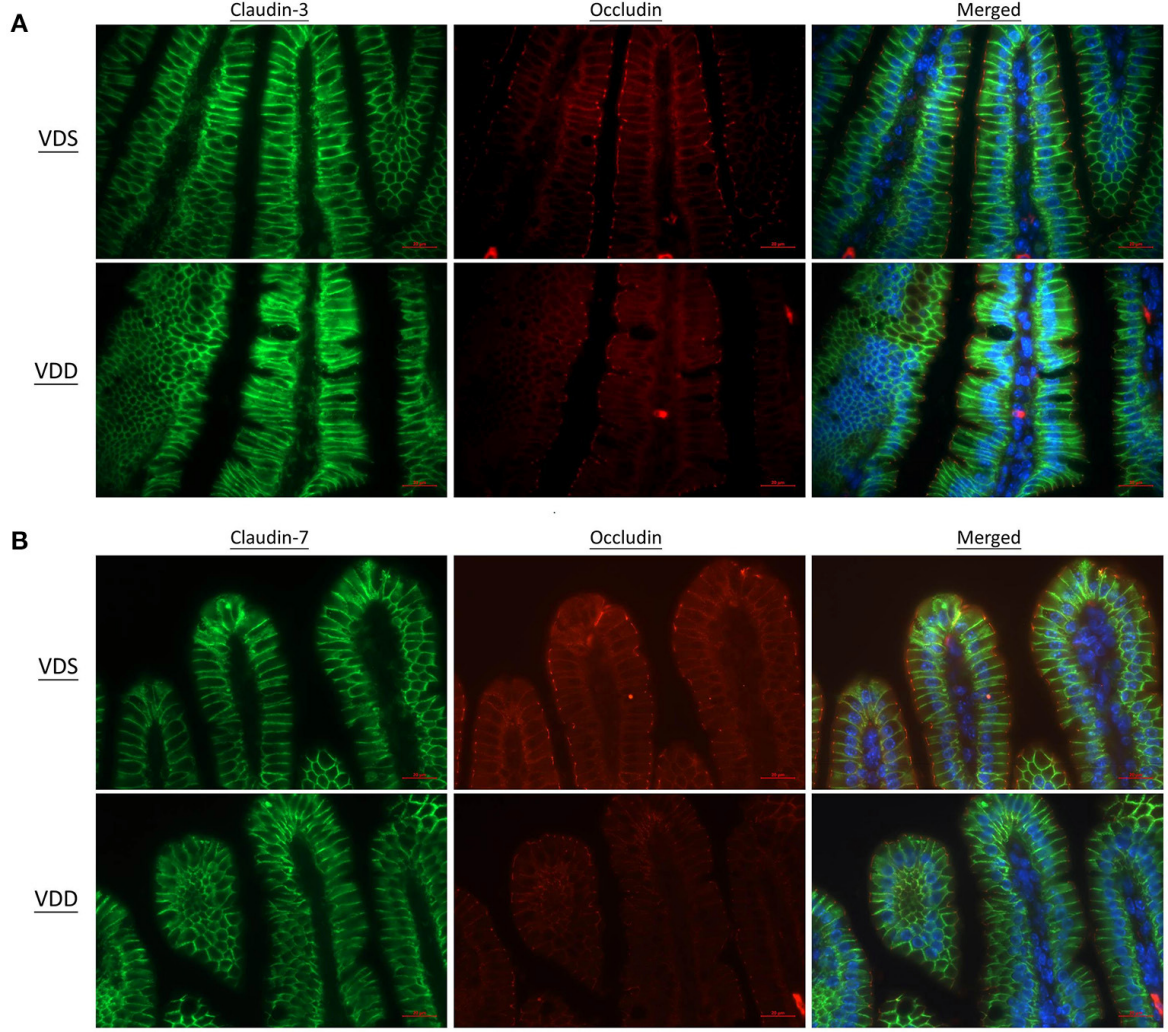
Vitamin D maintains intestinal tight junction expressions in VDD mice by fluorescence microscopy of the jejunum. DAPI was used for nucleus labeling (blue). Distribution of tight junctions was visualized by the claudin (CLD)-3 (DyLight 488, green) and occludin (OCDN) (DyLight 549, red) fusion proteins. CLD-3, OCDN, and DAPI were merged. **(A)** CLD-7 (DyLight 488, green), OCDN, and DAPI were merged. **(B)** The fluorescence line was clear and sharp in the VDS group but blurry, cloudy, and irregular in the VDD group at CLD-3, CLD-7, and OCDN distributions.

The effects of VDD on the jejunal TJ proteins' (ZO-1, CLD-1, CLD-3, CLD-7, and OCDN) structure and distribution are shown in Figures 5, 6. Immunofluorescence studies can provide general information on specific proteins (in this case ZO-1, CLD-1, CLD-3, CLD-7, and OCDN) concerning their overall pool (thickness of the signal) and distribution. A sharp signal means accumulation in specific cellular sites like the cell boundaries, suggesting that the structure of TJ is maintained, while a blurry signal suggests a more diffuse distribution within the cell cytoplasm and implies a dysfunction of TJ. In our study, the fluorescence lines for ZO-1, CLD-3, and OCDN proteins staining were clear and sharp in the VDS group but blurry, cloudy, and irregular in the VDD group (Figures 5A, 6A). Cell nuclei were found located at the baseline of enterocytes in the VDS group, but irregular arrangements of cell nuclei were observed in the VDD group. Similar findings were observed in CLD-1 and CLD-7 staining (Figures 5B, 6B). The surface of villi was straight and smooth in the VDS group but winding and irregular in the VDD group. OCDN was demonstrated rich in the apical villi in the VDS group as shown in the merged figure (Figure 6). However, we noticed that the colonic specimen texture in the VDD group was less elastic when compared with the VDS group while mice were sacrificed in this study. We compared the morphology of the colon to the jejunum and noticed that there were no obvious typical irregular edge and lining of the colon mucosa as we demonstrated in jejunal tissue.

### VDD Affects Intestine TJ Protein Expression

**Figure 7:**
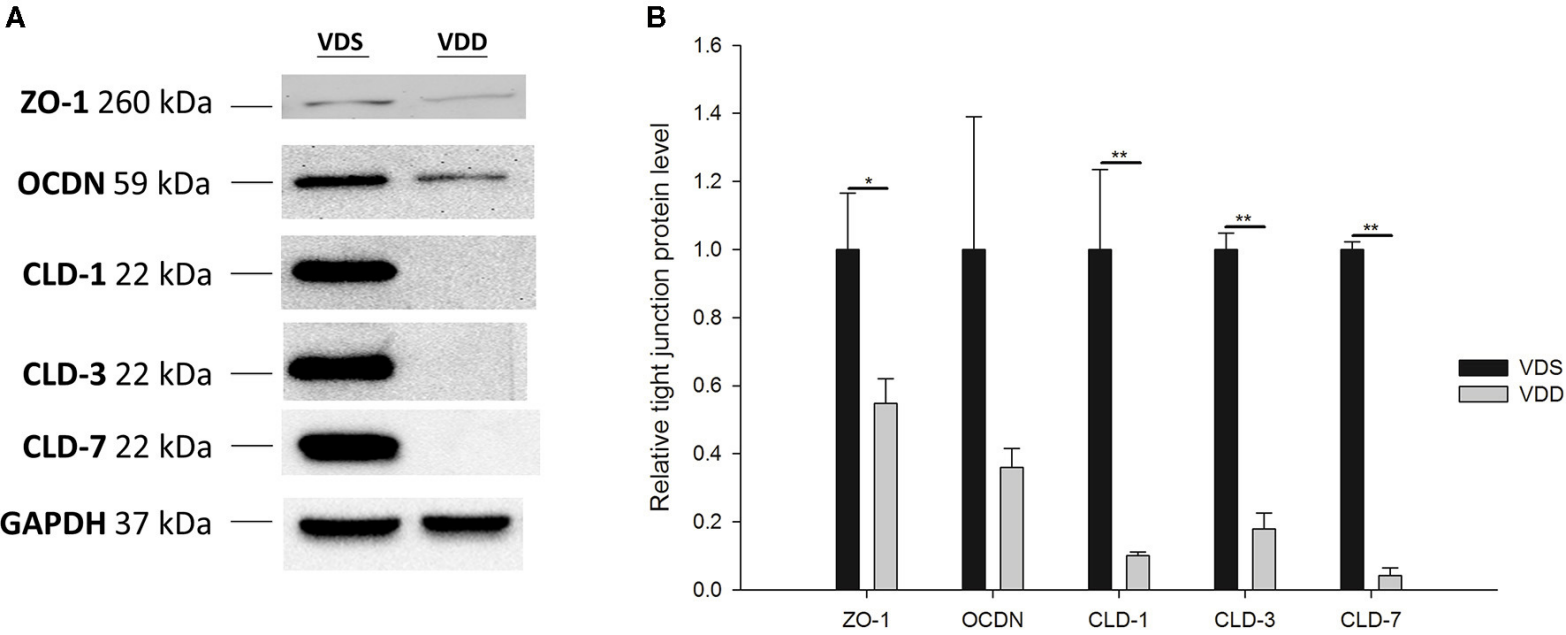
Effects of vitamin D deficiency on the jejunum tight-junction protein (CLD1, CLD3, CLD7, OCDN, and ZO-1) expressions. **(A)** Representative tight-junction protein levels by Western blot analysis. **(A)** The lines indicated the positions of CLD1, CLD3, CLD7, OCDN, and ZO-1, respectively. GAPDH served as a control of protein lysate loading. **(B)** The band densities of various tight-junction proteins were quantified by Image Lab^™^ software. All relative levels of CLD1, CLD3, CLD7, and ZO-1 protein expressions were significantly decreased in the VDD group when compared to the VDS group. No significant difference in relative level of OCDN was found between the two groups (*p* = 0.200). Values were analyzed using the independent *t*-test. (^*^p < 0.05, ^**^p < 0.001). Four mice in each group for parameter determination.

The effects of VDD on the jejunal TJ protein expression are shown in Figure 6. All relative levels of CLD-1 (VDD 0.10 ± 0.01 vs. VDS 1.00 ± 0.23, *p* = 0.007), CLD-3 (VDD 0.18 ± 0.05 vs. VDS 1.00 ± 0.05, *p* < 0.001), CLD-7 (VDD 0.04 ± 0.02 vs. VDS 1.00 ± 0.02, *p* < 0.001), and ZO-1 (VDD 0.55 ± 0.07 vs. VDS 1.00 ± 0.17, *p* = 0.038) protein expressions were significantly decreased in the VDD group when compared to the VDS group. However, we found no significant difference in the relative level of OCDN between the two groups (Figures 7A,B).

### VDD Upregulates Serum Inflammatory Cytokine Expressions

**Figure 8:**
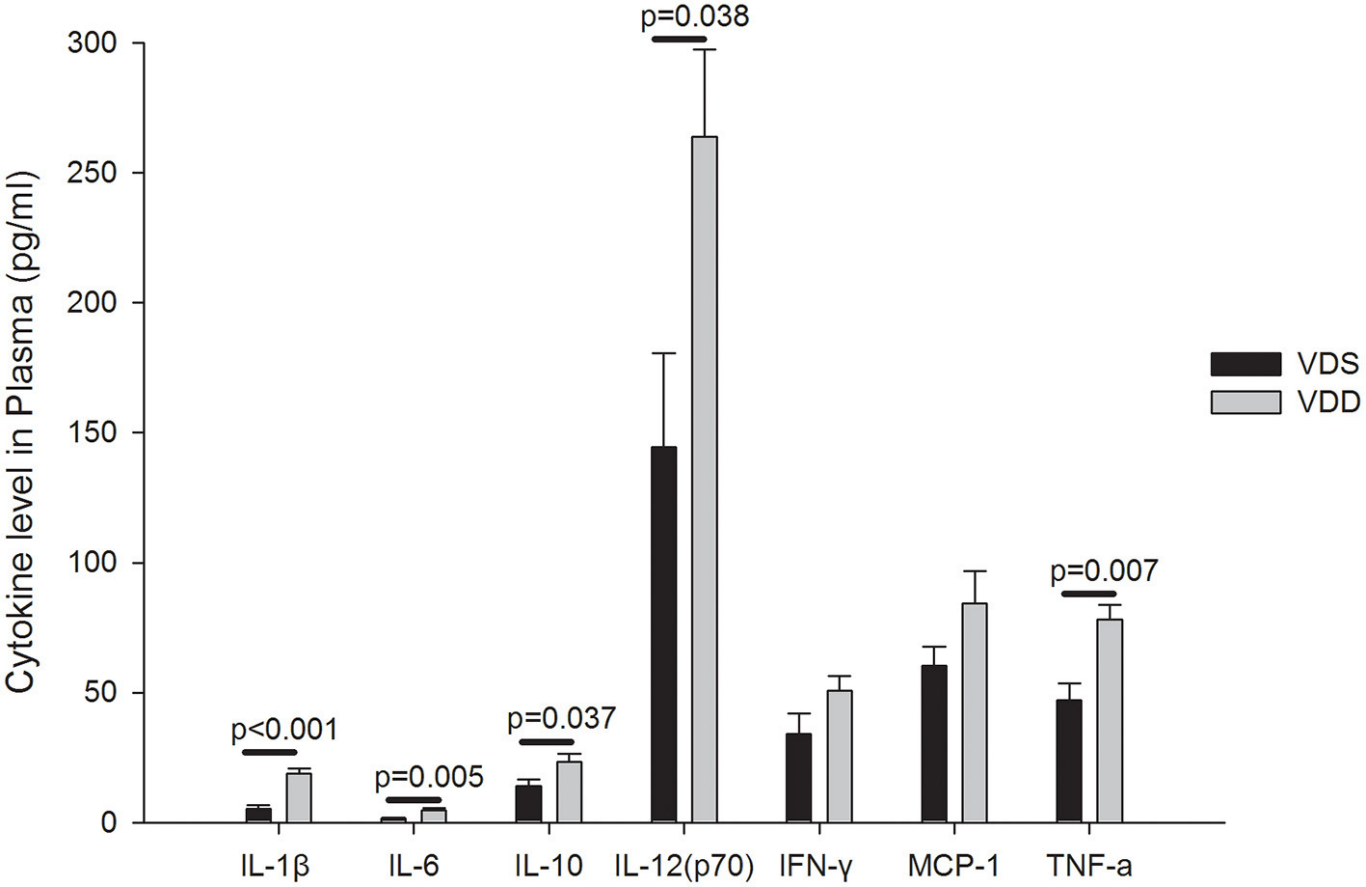
Effects of vitamin D deficiency on the plasma inflammatory cytokine expression. Upregulations of IL-1β, IL-6, IL-10, IL-12, and TNF-α were found in VDD mice. All these plasma inflammatory cytokines were significantly higher in the VDD group when compared to the VDS group. *p*-values were shown inside the figure. No significant differences in IFN-γ (*p* = 0.116) and MCP-1 (*p* = 0.119) were found between the two groups. Statistical analysis was performed by the independent *t*-test. Four mice in each group for parameter determination.

The effects of VDD on the serum inflammatory cytokine expressions are shown in Figure 8. Upregulations of IL-1β (VDD 20.55 ± 1.96 pg/ml vs. VDS 6.31 ± 2.17 pg/ml, *p* < 0.001), IL-6 (VDD 5.93 ± 1.00 pg/ml vs. VDS 1.75 ± 0.36 pg/ml, *p* = 0.005), IL-10 (VDD 26.84 ± 4.42 pg/ml vs. VDS 25.36 ± 4.10 pg/ml, *p* = 0.037), IL-12 (VDD 281.43 ± 40.94 pg/ml vs. VDS 143.23 ± 44.98 pg/ml, *p* = 0.038), and TNF-α (VDD 82.53 ± 8.05 pg/ml vs. VDS 64.91 ± 12.58 pg/ml, *p* = 0.007) were found in VDD mice. All these serum inflammatory cytokines were significantly higher in the VDD group when compared to the VDS group. However, we found no significant differences in IFN-γ and MCP-1 between the two groups.

### VDD Affects the Serum and Intestinal Zonulin Levels

**Figure 9:**
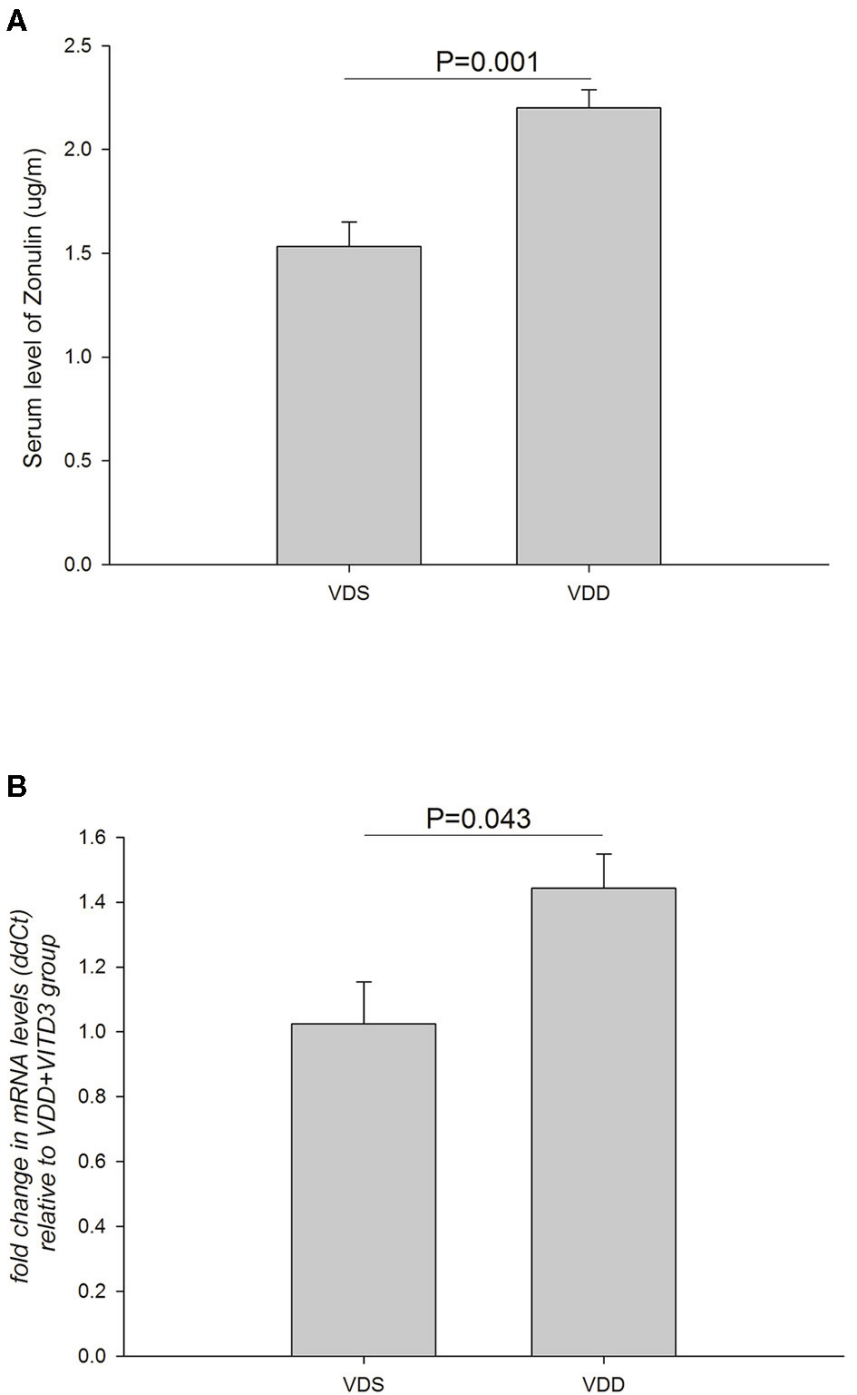
Effects of VDD on the serum and intestinal zonulin levels. **(A)** Serum zonulin levels between the VDD and VDS groups. A significant difference in the serum zonulin level was found (*p* = 0.001). **(B)** A significantly higher level of mRNA expression of jejunum zonulin was observed in the VDD group (*p* = 0.043). Statistical analysis by independent *t*-test. Four mice in each group for parameter determination.

The effects of VDD on the serum zonulin level and intestinal zonulin expression are shown in Figure 9. A significant difference in serum zonulin levels was found between the two groups (VDD 2.20 ± 0.09 μg/ml vs. VDS 1.53 ± 0.12 μg/ml, *p* = 0.001) (Figure 9A). Similarly, a significantly higher relative level of mRNA expression of jejunum zonulin was observed in the VDD group (VDD 1.44 ± 0.11 vs. VDS 1.03 ± 0.13, *p* = 0.043) (Figure 9B).

## Discussion

VD has demonstrated multifaceted effects on gut health and has been shown to target three major components of the gastrointestinal tract: intestinal epithelial barrier, gut immunity, and gut microbiota (30). Previous studies had shown that VDD was strongly correlated with gut integrity and immune response (12, 31). However, the mechanisms underlying the protective effects of VD on intestinal barrier function remain essentially unclear. In this study, we successfully established an animal model by raising C57BL/6 wild-type mice fed the VDD diet and elucidated the roles of VD on gut morphology and barrier functions. C57BL/6 wild-type mice were useful and popular mouse strains in gastrointestinal tract studies (32, 33). In our study, the serum level of VD was significantly reduced in the VDD group when compared with the VDS group and showed a deficient level according to human criteria. We also investigated the enterocyte microstructures, inflammatory cytokines, and TJ protein expressions with promising results. For the first time, we successfully demonstrated that VDD diet could lead to a significant upregulation in the mRNA expression of the jejunum zonulin level in a mouse model.

### Effects of VDD on Jejunum and Colon Histology

In this mouse model, we demonstrated that VD did have a protective effect in the development of intestinal epithelial and colonic cells. We found that a typical irregular edge and lining of the jejunal mucosa and distorted enterocytes with significant inflammatory cell infiltration causing mucositis were revealed in VDD mice. However, the intestinal villi of the mouse in the VDS group were much uniform and no inflammatory cell infiltrations were found. We noticed that villus height, crypt depth, and villus height/crypt depth ratio of the jejunum were similar in both groups and no significant differences were found. Similarly, histological damages were found in the colonic histology in the VDD group, including crypt hyperplasia, loss of epithelial integrity, and inflammatory cell infiltration. VDD mice had significantly decreased colonic mucosa thickness compared to mice raised on VDS diet after 7 weeks.

Besides distorted morphology, VDD caused significant intestinal inflammation compared to the control group in our study. Ryz et al. observed that VDD mice showed more infiltrating macrophages and neutrophils in the cecal tissues, particularly in the submucosal regions when compared with VDS mice (21). In a previous mouse model study, Assa et al. demonstrated a hyperplastic response in sham-infected VDD mice when compared with sham-infected VDS mice (12). Wang et al. also found that proliferation and apoptosis of intestinal epithelial cells played critical roles in cirrhosis-associated intestinal mucosal barrier dysfunction (34). Their results showed that VD restored the proliferative ability of crypt cells in the intestines, inhibited enterocyte apoptosis, maintained the normal intestinal epithelial turnover, and improved the integrity and function of the intestinal epithelial barrier in CCl_4_-induced liver cirrhotic rats.

### Effects of VDD on Intestine TJ Proteins Structure and Expression

We showed that the fluorescence lines for ZO-1, CLD-1, CLD-3, CLD-7, and OCDN protein staining were clear and sharp in the VDS group but blurry, cloudy, and irregular in the VDD group. Cell nuclei were found located at the baseline of enterocytes in VDS mice, but irregular arrangements of cell nuclei were observed in the VDD group. Besides, levels of CLD-1, CLD-3, CLD-7, and ZO-1 proteins were significantly decreased in the VDD group when compared to the VDS group. Zhao et al. demonstrated that when compared to TJ expressions in the control group, there were significantly reduced expressions in ZO-1, OCDN, and CLD-1 in the VDD group (13). They also found that TJ marker expressions in the VD-treated group were also significantly higher than those in the VDD group, which suggested the importance of VD to maintain the integrity of the TJ complex. Consistent with previous studies, our findings demonstrated that significant intestinal morphological alterations and TJ protein loss occurred in VDD mice. We also showed that ZO-1, CLD-1, CLD-3, CLD-7, and OCDN were highly expressed in jejunum tissues in the VDS group.

However, not all the research studies demonstrated beneficial effects of VD on intestinal barrier function, Mandle et al. in their randomized controlled trial concluded that no evidence was found for incremental effects of supplemental VD on CLD-1, OCDN, and MUC12 levels in the normal colorectal mucosa of patients at increased risk for colorectal cancer (35). Their findings do not support that VD alone substantially affects the expression of the three biomarkers.

### Effects of VDD on the Serum Inflammatory Cytokine Expressions

One of the most significant impacts of VD in our study was its effect on the host inflammatory response within the intestine. Even under uninfected conditions, VDD mice showed a higher intestinal inflammatory condition with elevated intestinal expressions of various pro-inflammatory cytokines. Soares et al. suggested two principles of mucositis development including firstly the generation of reactive oxygen species which directly damaged cells, tissue, and blood vessels and secondly the upregulation of pro-inflammatory cytokines including TNF-α, IL-1β, and IL-6 which caused further mucosal injuries (36). In our mouse model, all measured serum cytokines except IFN-γ and MCP-1 were significantly higher in the VDD group when compared to the VDS group. We showed that VD played important roles in the immune modulation and processed anti-inflammatory effects. VD has been shown to modulate a wide variety of immune responses. Assa et al. found that VD exerted its effect on the host inflammatory response within the intestine. VDD mice had elevated expressions of IL-17A and IL-17F in the distal colon compared with VDS mice (12). Uninfected VDD mice expressed higher mRNA transcripts for both pro- and anti- inflammatory cytokines in colonic homogenates. Blaschitz et al. reviewed that local immune responses serve to contain infections by pathogens to the gut while preventing pathogen dissemination to systemic sites. Several subsets of T cells in the gut contribute to the mucosal response to pathogens by secreting a subset of cytokines including IL-17A, IL-17F, IL-22, and IL-26. These cytokines induce the secretion of chemokines and antimicrobial proteins, thereby orchestrating the mucosal barrier against gastrointestinal pathogens (37). Additionally, IL-1β also plays a crucial role in the activation of the NF-kB pathway, even working with TNF for a synergistic effect in kickstarting the inflammatory response of endothelial adhesion molecules (38). Proinflammatory cytokines including TNF-α, IL-1β, and IL-6 were shown to play important roles in amplifying the severity of chemotherapy-induced intestinal mucositis (39). In our previous study, we demonstrated that those mice in the 5-FU-induced intestinal mucositis group had significantly higher levels of circulating pro-inflammatory cytokines which decreased significantly after probiotic administration (26). We found similar results in using SCID/NOD mice as animal model, suggesting that innate immunity plays a role in the pathogenesis of intestinal mucositis (40). It seems that VD also exerts similar anti-inflammatory effects through the inhibition of pro-inflammatory cytokine expressions according to the results of this mouse model study.

### Effects of VDD on the Serum and Intestinal Zonulin Levels

In the recent decade, Fasano et al.'s serial studies led to the discovery and characterization of zonulin as the only human protein discovered to date that is known to reversibly regulate intestinal permeability by modulating intercellular tight junctions (41–43). They have generated evidence that the small intestine exposed to enteric bacteria secreted zonulin (44). Following the release of zonulin, the intestine showed increased permeability (leaky gut) and disassembly of ZO-1 from the TJ complex (45). A systematic review of the literature revealed that zonulin has been reported as a biomarker of several pathological conditions, including autoimmune diseases, diseases of the nervous system, and neoplastic conditions (44, 45).

Emerging data have led to the hypothesis that VD plays a role in promoting epithelial barrier function. However, the relationship of VD and serum and intestine zonulin levels was seldom discussed. In a prospective study to document the relationship between the admission vitamin D deficiency and markers of intestinal permeability in hospitalized patients who were critically ill, Eslamian et al. showed that median plasma endotoxin and zonulin decreased with increasing serum levels of VD categories in the overall study population (46). Their finding suggested a relationship between VDD and early alterations in intestinal permeability. Increased intestinal permeability causing a leaky gut phenomenon has been shown to play a crucial role in the pathogenesis of IBDs. In humans, serum and fecal zonulin were found to be elevated in patients with active Crohn's disease but not with ulcerative colitis (47). In a recent study, serum zonulin concentration was found to be higher in both diseases, and an inverse correlation was observed between serum zonulin concentration and disease duration (48).

In this study, we looked at the effects of VDD on the serum and intestine zonulin levels. We observed that there was a significantly higher level of mRNA expression of jejunum zonulin in the VDD group. Similarly, a marked increase in serum zonulin level was found in the VDD group. Our findings suggested that VDD diet did induce mucosal barrier dysfunction and initiate the release of zonulin in the jejunum. Mucosal injury thus caused a significant rise in serum zonulin level in this mouse model. Asmar et al. stated that zonulin is usually triggered to release when the small intestine is exposed to enteric pathogens and gluten (42). Our findings suggested that zonulin could be released whenever there was mucosa barrier injury or leaky gut conditions even in a non-infected VDD mouse model. We also demonstrated that serum zonulin could reflect the level of zonulin in the intestinal tract and assessment of the serum zonulin level is desirable and clinically more feasible. Whether the fecal zonulin level correlates with the severity of intestinal mucosa injury was not studied in our study but warrants further investigation.

However, we recognize that zonulin is secreted not only from enterocytes; it has been found in several extra-intestinal tissues, e.g., adipose tissue, brain, heart, immune cells, liver, lungs, kidney, and skin (49, 50). Thus, the levels of zonulin in serum reflect not only intestinal secretion but also secretion from other organs. Nevertheless, to date it is impossible to elucidate the exact origin of the serum zonulin level and the studies on the roles of zonulin in extra-intestinal tissues are limited. Serum zonulin levels are supposed to reflect mainly the intestinal permeability and act as a marker of gut integrity.

Studies on the association of intestinal zonulin expression and TJ composition are few in the literature. Feng et al. demonstrated that dietary bisphenol A (BPA) uptake destroys the morphology of the colonic epithelium and increases the pathology score (51). The levels of endotoxin, diamine peroxidase, D-lactate, and zonulin are significantly elevated in both plasma and colonic mucosa. The expression of TJ proteins (ZO-1, occludin, and claudin-1) in the colonic epithelium of BPA mice decreased significantly, and their gene abundance was also inhibited.

### Roles and Mechanisms of VD on TJ Proteins and Gut Integrity

TJ are the most apical junctional complex connecting both neighboring epithelial and endothelial cells. They comprised various transmembrane proteins. VD plays a crucial role in protecting the integrity of the intestinal epithelial barrier against infectious and inflammatory insults. Gubatan et al. have suggested that VD can enhance innate immunity by inducing antimicrobial peptides and regulate adaptive immunity by promoting anti-inflammatory T cells and cytokines (30). Besides, Kong et al. have proved that VDD may compromise the mucosal barrier, leading to increased susceptibility to mucosal damage and increased risk of IBD (11). An increased permeability in the TJ may provide a major site for both infection and establishment of inflammation in the gut (52–54). Bacterial translocation is believed to occur *via* a paracellular pathway through the epithelial cells causing a leaky gut phenomenon.

VD was found to be able to protect the intestinal barrier from injuries induced by multiple reagents (13, 39, 55). Several mechanisms by which VD exerts anti-inflammatory effects have been suggested. Using Caco-2 monolayers as *in vitro* models and a gluten-sensitized mouse model as an *in vivo* model, Dong et al. recently investigated the protective effect of 1,25-dihydroxyvitamin D3 on pepsin–trypsin-resistant gliadin-induced tight-junction injuries (56). They successfully demonstrated that, both *in vitro* and *in vivo*, VD3 significantly attenuated the TJ injury-related increase in intestinal mucosa barrier permeability. VD3 treatment upregulated the TJ protein expression levels and significantly decreased the MyD88 expression and zonulin release signaling pathway.

Zhao et al. showed that VD might have a protective effect on barrier integrity by maintaining the expression of TJ proteins, thereby reducing the severity of gut inflammation (13). Using the VDD mouse model, Zhang et al. successfully demonstrated that both the differentiation of Th1 cells and the production of relative cell cytokines (IL-2, IFN-γ, and TNF-β) were inhibited by 1,25(OH)_2_D_3_. The addition of 1,25(OH)_2_D_3_ has direct effects on CD4+ T cells and supports its potent immunosuppressive benefits in the treatment of a number of other autoimmune diseases (57). Other studies have shown that VD protects the gut epithelial barrier by suppressing gut epithelial cell apoptosis (58, 59). In this mouse model, our data showed that VD might have a protective effect on barrier integrity by maintaining the expression of TJ proteins, thereby reducing the severity of gut inflammation.

This study adds to previous reports that the bioavailability of VD is an important contributing factor for determining the epithelial integrity. Once the mucosal barrier is breached, the submucosa is exposed to a vast pool of luminal antigens, including food and bacteria, thereby engaging the innate immune responses including increased production in proinflammatory cytokines TNF-α and IFN-γ. We demonstrated that VDD caused a significant destruction of the intestinal morphology and an increase in the circulating pro-inflammatory cytokines such as IL-1, IL-6, TNF-α, and IFN-γ. The reduction in these cytokines by VD may be either due to its direct suppressive effect on the expression of these pro-inflammatory cytokines or due to the effect on maintenance of epithelial barrier function, leading to a reduction in foreign luminal antigenic load, and a full activation of the innate immune system.

In summary, our data suggest that VD may be effective in the maintenance of gut integrity, decreasing histological inflammation, enhancing epithelial cell resistance to injury, and suppressing pro-inflammation responses to luminal antigens. Previous studies seldom emphasized and investigated the role of VD on gut morphology and TJ proteins. In this study, we successfully established an animal model by raising mice fed a VDD diet to elucidate the roles of VD on gut morphology and barrier function. To our knowledge, this is the first study to demonstrate that VDD could lead to a significant upregulation in mRNA expression of jejunum zonulin in a mouse model.

There are several limitations in our study. One limitation is that the study period was indeed short and the observation of possible pathological changes might be inadequate. Various doses of VD supplementation were not assessed to determine their possible contributions to the observed effects. Another limitation is that we did not address the possible mechanisms by which VD exert their beneficial outcomes such as the effects on intestinal permeability and transepithelial electrical resistance and the influence on the composition and diversity of the intestinal microbiota. These areas should be investigated in future experiments. Besides, we only assess the TJ proteins ZO-1, CLD-1, CLD-3, CLD-7, and OCDN in this study. The expressions and roles of other TJ proteins such as pore-forming CLD-2 and−15 warrant further investigation. In this study, we do not investigate the roles of microbiota and the consequences of dysbiosis that may probably occur in VDD mice. It would be also helpful to complement our study with the gene expression of the same TJ proteins to establish whether VDD causes a decreased gene expression of these proteins or an increased degradation of their protein pool. Nevertheless, the greatest challenge for an animal model is the difficulty in translating results obtained from the current model to the wide range of human patient groups with varying ages and diagnoses. More clinical works are needed to demonstrate the beneficial effects of VD and to elucidate the correct dosing regimens for the management of various human disorders with intestinal barrier dysfunction.

## Conclusions

We successfully demonstrated that VDD could lead to impaired barrier properties. We assume that sufficient VD could maintain intestinal epithelial integrity and prevent mucosal barrier dysfunction. Thus, VD supplementation may serve as part of therapeutic strategy for human autoimmune or infectious diseases with intestinal barrier dysfunction (leaky gut phenomenon) in the future. To our knowledge, this is the first study to demonstrate that VDD could lead to a significant upregulation in mRNA expression of jejunum zonulin level and also a marked elevation of serum zonulin in a mouse model.

## Data Availability Statement

The original contributions presented in the study are included in the article/supplementary material, further inquiries can be directed to the corresponding author/s.

## Ethics Statement

The animal study was reviewed and approved by the Institutional Animal Care and Use Committee (IACUC) of MacKay Memorial Hospital (IACUC number: MMH-A-S-107-026).

## Author Contributions

C-YY, W-TC, J-SC, C-YL, C-WC, and H-CL conceived and designed the experiments. C-YY, M-LC, and J-SC performed the experiments. C-YY, W-TC, C-BJ, S-WC, M-LC, and C-YL analyzed the data. C-YY, W-TC, C-BJ, S-WC, M-LC, C-YL, C-WC, and J-SC contributed the reagents/materials/analysis tools. C-YY, W-TC, C-BJ, S-WC, M-LC, C-YL, J-SC, C-WC, and H-CL wrote the paper. All authors contributed to the article and approved the submitted version.

## Conflict of Interest

The authors declare that the research was conducted in the absence of any commercial or financial relationships that could be construed as a potential conflict of interest.

## Publisher's Note

All claims expressed in this article are solely those of the authors and do not necessarily represent those of their affiliated organizations, or those of the publisher, the editors and the reviewers. Any product that may be evaluated in this article, or claim that may be made by its manufacturer, is not guaranteed or endorsed by the publisher.
